# Investigation of diabetic patient’s fingernail quality to monitor type 2 diabetes induced tissue damage

**DOI:** 10.1038/s41598-019-39951-3

**Published:** 2019-02-28

**Authors:** Praveer Sihota, Ram Naresh Yadav, Vandana Dhiman, Sanjay Kumar Bhadada, Vishwajeet Mehandia, Navin Kumar

**Affiliations:** 1 Department of Mechanical Engineering, Indian Institute of Technology (IIT) Ropar, Rupnagar, Punjab 140001 India; 2 Department of Endocrinology, Post Graduate Institute of Medical Education and Research (PGIMER), Chandigarh, 160012 India

## Abstract

Long-term Type 2 Diabetes (T2D) affects the normal functioning of heart, kidneys, nerves, arteries, bones, and joints. The T2D gradually alters the intrinsic material properties, and structural integrity of the tissues and prolonged hyperglycemia causes chronic damages to these tissues quality. Clinically no such technique is available which can assess the altered tissues quality associated with T2D. In the present study, the microstructural characterization (surface morphology, surface roughness and density and calcium content), material characterization (modulus, hardness), and macromolecular characterization (disulfide bond content, protein content and its secondary structure) are investigated among healthy, diabetic controlled (DC) and uncontrolled diabetic (UC) group of fingernail plate. It is found that T2D has an adverse effect on the human fingernail plate quality. The parameters of nail plate quality are changing in a pattern among all the three groups. The properties mentioned above are degrading in DC group, but the degradation is even worst in the case of severity of T2D (UC group) as compared to the healthy group (Healthy<DC<UC). This study suggests that the fingernail plate quality has a potential to become a new avenue to assess the secondary diabetic complications, i.e. to assess the bone quality.

## Introduction

Type 2 Diabetes (T2D) is characterized by high blood glucose level resulting from Insulin resistance and/or deficiency^1^. According to International Diabetes Federation (IDF) 2015, 415 million adults (age range 20–79 years) have diabetes worldwide, among them India is home to a second largest number of diabetes cases (69.2 million in 2015)^2^. Predicted data shows that by 2040 the prevalence of diabetes will rise to 642 million adults worldwide and among them, 123.5 million will be in India^2–5^. IDF 2015 also reported that around 192.8 million people all over the world are undiagnosed for T2D^2,6,7^. Above data shows that the diabetes is a global public health problem.

The T2D is diagnosed based on the elevated blood glucose and the HbA1c (Hemoglobin A1c, glycated hemoglobin) level^8^. The HbA1c is a reliable representation of long-standing uncontrolled blood glucose (hyperglycemia)^8^. The long-standing hyperglycemia is prone to increase the non-enzymatic reactions (Maillard reactions) and form advanced glycation end-products (AGEs)^9–12^. The AGEs are a category of post-translation modification^13^, and they are highly cross-linked & slightly soluble^9^. Among a diverse group of compounds, one of the best-studied AGE is carboxymethyl-lysine (CML)^14,15^. The AGEs get accumulated into the tissues and react irreversibly with amino acid residues of peptides or proteins to form protein adducts (protein – AGE) or protein crosslinks (AGE-protein-AGE)^16^. This phenomenon is widely recognized as the major cause of secondary diabetic complications, as it alters the tissue quality and its normal functioning, i.e., heart, kidneys, nerves, arteries, lens, tendons, skin, bones, and joints^10,11,17–26^.

Clinically there is a lack of suitable techniques which can assess or monitor the general tissue damage associated with T2D. Tissue damage is a catastrophic event, and one of the early diagnoses can be the monitoring of adverse changes in the tissue quality. For testing the tissue quality *in vitro*, the surgeon needs to do biopsy, which is painful, invasive, and involves the risk of infection or slow healing. Therefore, the keratinized epithelial tissue such as fingernail plate is a useful site to monitor the general tissue damage, because the major constituent of fingernail plate (Keratins, present in ± 85%) is also prone to glycation^10,27,28^. Interestingly, the growth of the nail plate is slow, hence it is a particularly important material to evaluate the long-term effects of hyperglycemia on the tissue quality^24^. Additionally, this monitoring technique is painless, non-invasive, and it is also economical because it does not consume expensive reagents. After considering the advantages to study the fingernail plate quality, we have explored the available literature on fingernail plate, and it is best inferred that comprehensive research is still lacking on the effect of T2D on fingernail plate quality.

**Figure 1:**
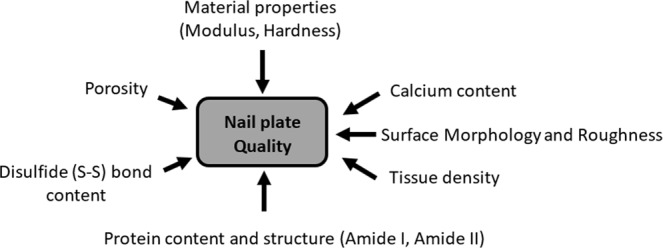
Determinants of nail quality.

The major parameters that contribute to nail plate quality **(**Fig. 1**)** are, the nail surface morphology and roughness, tissue density, mineral content, material properties, disulfide bond content and protein composition and structure^29^. In this study, we have investigated the above nail plate quality parameters for healthy, diabetic controlled (DC) and uncontrolled diabetic (UC) groups of fingernail plate.

## Results

### Patient’s characteristics

The distribution of male and female are found 40% and 60% respectively in all three groups as mentioned in Table 1. The mean HbA1c levels are found significantly high for DC and UC groups 6.6 (6.2–7.3)%, and 8.4 (7.7–14)% respectively as compared to healthy 5.4 (5.1–5.9)% as shown in Table 1.

**Table 1:** Clinical details of fingernail specimens.

Group no	Group name	Gender Distribution		Age (years)	Average HbA1c (%)
		Male	Female		
1	UC (uncontrolled diabetic)	10	15	60 (42–70)	8.4 (7.7–14)***
2	DC (diabetic controlled)	8	12	55 (40–77)	6.6 (6.2–7.3)**
3	Healthy	12	18	58 (42–77)	5.4 (5.1–5.9)

**p < 0.01 and ***p < 0.001 compared to healthy group.

### Porosity

**Figure 2:**
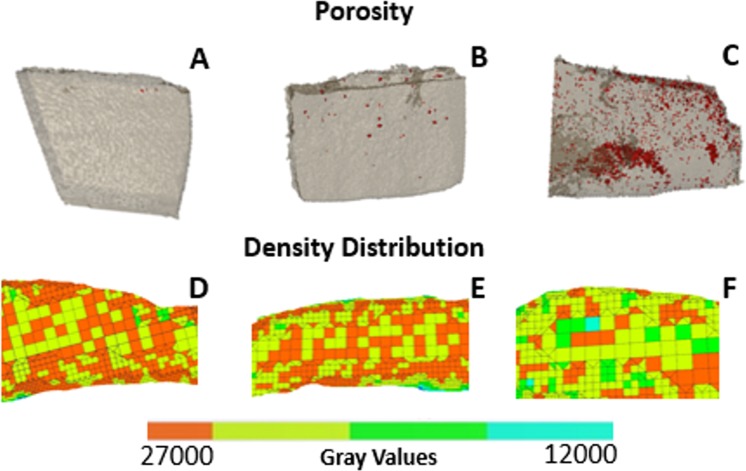
Micro CT images of fingernail plate samples describing porosity and density distribution in healthy, diabetic controlled (DC), and uncontrolled diabetic (UC) groups (**A**,**D**- healthy, **B**,**E** -DC, **C**,**F**- UC).

**Figure 3:**
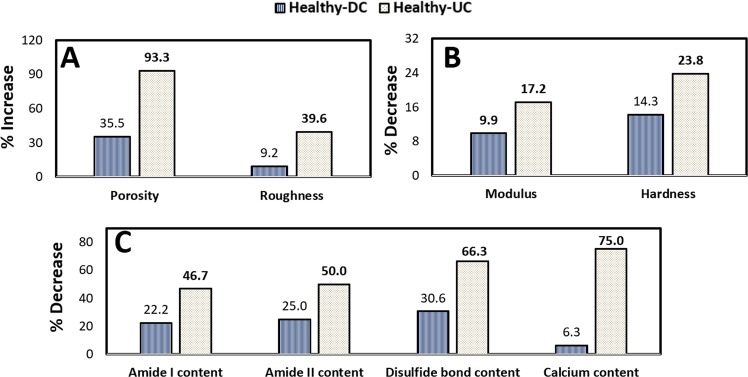
Percentage change in the nail plate quality parameters for diabetic controlled (DC) group (Healthy-DC) and uncontrolled diabetic (UC) group (Healthy-UC) with respect to healthy group (**A**) The percentage increase in porosity and roughness (structural properties), (**B**) Percentage decrease in modulus and hardness (material properties), (**C**) The percentage decrease in Amide I, Amide II, Disulfide bond and Calcium content (Biochemical properties). (In the figure the bold values are representing a significant change from the healthy group).

The fingernail plate porosity is shown with red color in Fig. 2(A–C) for healthy, DC and UC group respectively. The porosity is small in healthy group, moderate in DC group and high in UC group. The mean values of percentage porosity are presented in Table 2. The percentage increase in porosity is 35.5% in DC group and 93.3% (p < 0.001) in UC group with respect to healthy group as shown in Fig. 3(A). The density distributions are shown in Fig. 2(D–F) for healthy, DC and UC group respectively. The higher gray values in these figures (shown with orange color) indicate the presence of denser content (minerals), which is found high in healthy group, moderate in DC group and small in UC group.

**Table 2:** Nail quality parameters of the human fingernail.

Characterization techniques		Parameters studied	Study groups		
			Healthy	Diabetic controlled (DC)	Uncontrolled diabetic (UC)
Calcium content (EDXS)		Ca (weight %)	0.64 ± 0.04	0.60 ± 0.07	0.16 ± 0.04***
Tissue density		Density (g/cc)	1.31–1.35	1.28–1.32	0.99–1.20**
Micro structure (Nano CT)		Porosity (%)	0.020 ± 0.003	0.031 ± 0.007	0.298 ± 0.041***
Surface roughness	AFM	Roughness (Rq) (nm)	48.93 ± 4.12	53.88 ± 3.68	81.03 ± 4.31*
Material properties (Nanoindentation)		Modulus (GPa)	4.66 ± 0.08	4.20 ± 0.12*	3.86 ± 0.12***
		Hardness (GPa)	0.21 ± 0.05	0.18 ± 0.04	0.16 ± 0.05***
Macro molecular vibrations (FTIR)	Protein structure	Amide I position (cm^−1^)	1640.72 ± 3.69	1643.93 ± 2.88	1646.24 ± 3.31**
		Amide II position (cm^−1^)	1534.23 ± 3.21	1536.11 ± 3.77	1537.84 ± 1.30*
	Protein content	Amide I band area (arb. unit)	0.45 ± 0.16	0.35 ± 0.14	0.24 ± 0.08**
		Amide II band area (arb. unit)	0.36 ± 0.12	0.27 ± 0.09	0.18 ± 0.05**
	Disulphide (S-S) bond content	Peak height S-S peak/CH_2_ peak	0.98 ± 0.12	0.68 ± 0.11	0.33 ± 0.11***

*p < 0.05, **p < 0.01 and ***p < 0.001 respectively compared to healthy group.

### Surface morphology and roughness

**Figure 4:**
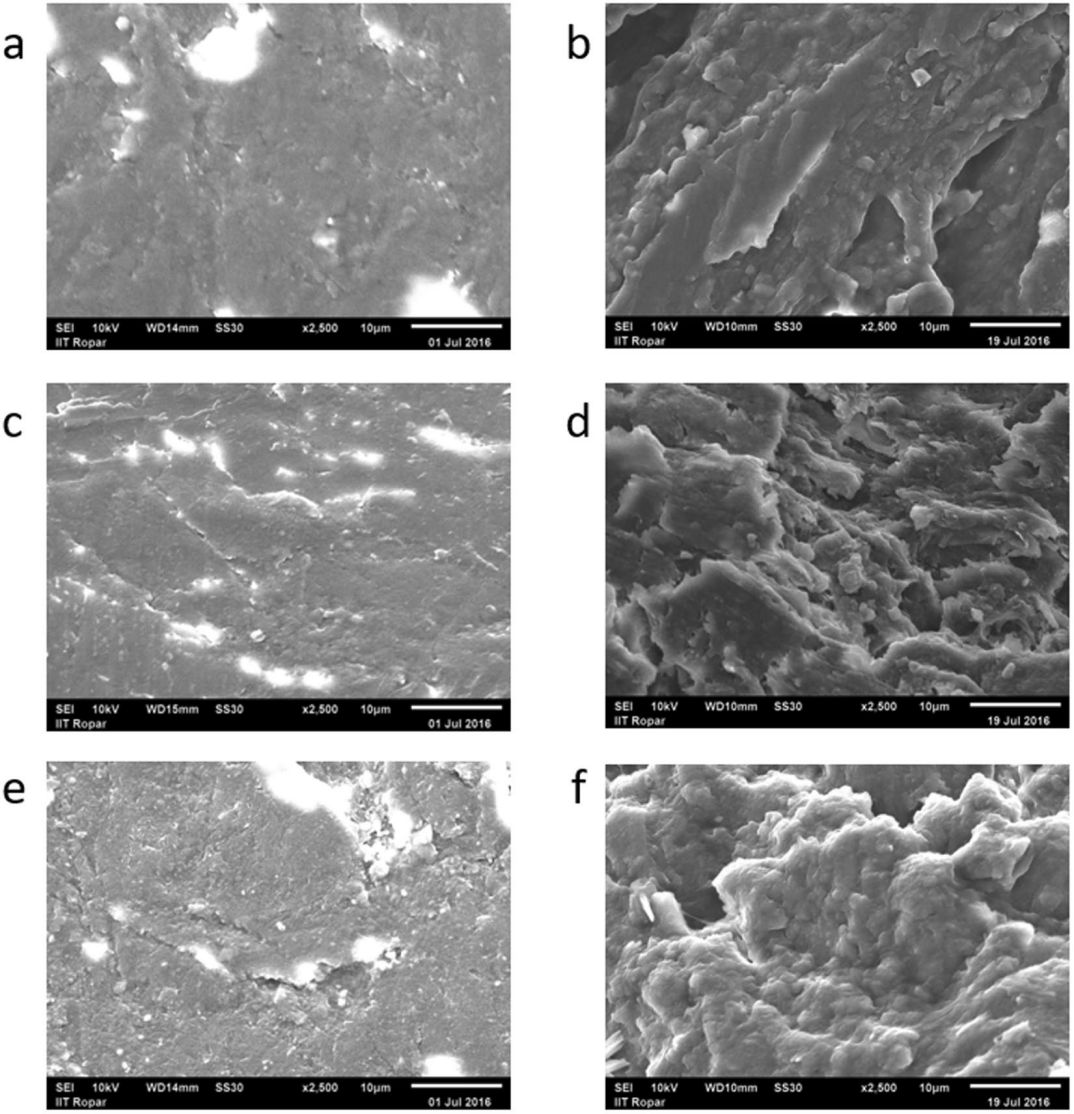
SEM images showing the morphology of fingernail plate for healthy, diabetic controlled (DC), and uncontrolled diabetic (UC) groups (**a**) Dorsal phase (healthy), (**b**) Ventral phase (healthy), (**c**) Dorsal phase (DC), (**d**) Ventral phase (DC), (**e**) Dorsal phase (UC), (**f**) Ventral phase (UC).

The SEM images of the dorsal and ventral phase (surface morphology) of clipped nail plate are shown in Fig. 4 for healthy, DC and UC samples. The results revealed that the surface morphology is moderately altered in DC group and highly altered in UC group as compared to the healthy group. In T2D groups, the surface morphology of the ventral layer is affected more than the dorsal layer because the bottom layer of the nail plate is more glycated than the upper layer as it remains in close contact with the blood vessels and the interstitial fluid^10^.

**Figure 5:**
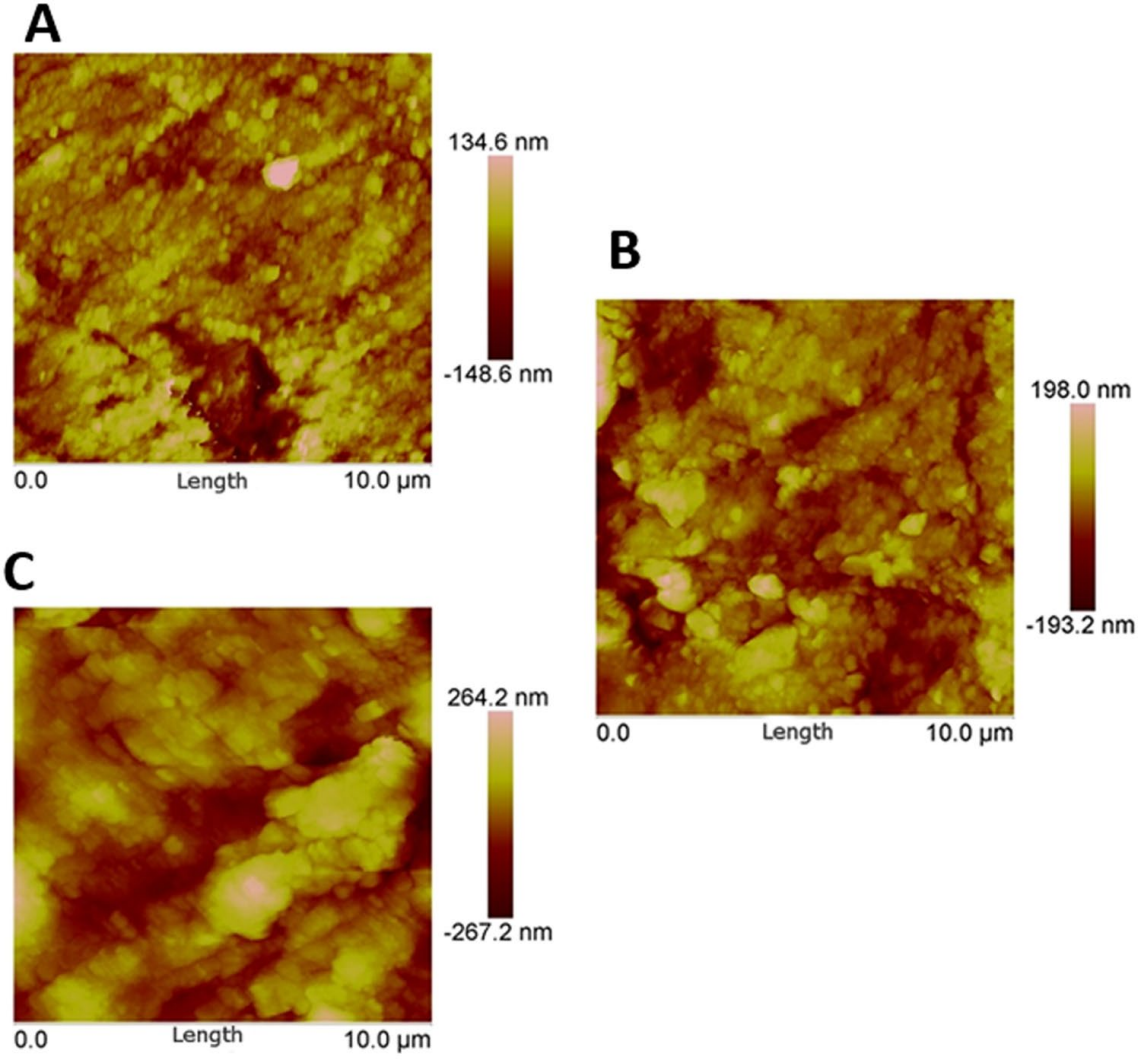
Surface roughness of Dorsal phase of fingernail plate obtained through AFM experiments (**A**) Healthy group, (**B**) diabetic controlled (DC) group, (**C**) uncontrolled diabetic (UC) group.

The surface roughness measured through AFM is shown in Fig. 5, and the root-mean-square value of roughness (Rq) obtained through these experiments are shown in Table 2. The percentage increase in surface roughness is 9.2% in DC group and 39.6% (p = 0.04) in UC group as shown in Fig. 3(A).

### Material properties

**Figure 6:**
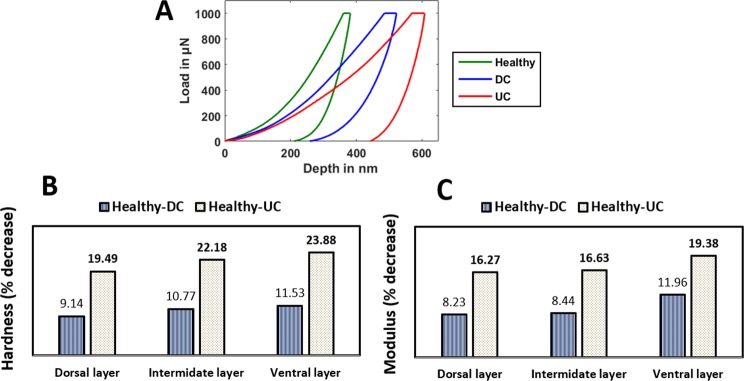
Nanoindentation results, (**A**) Representative load-displacement curves of healthy, diabetic controlled (DC) and uncontrolled diabetic (UC) group, (**B**) The percentage decrease in hardness for DC group (Healthy-DC) and UC group (Healthy-UC) with respect to healthy for dorsal, intermediate and ventral layer, (**C**) The percentage decrease in modulus for DC group (Healthy-DC) and UC group (Healthy-UC) with respect to healthy for dorsal, intermediate and ventral layer. (In the figure the bold values are representing the significant change with respect to the healthy group).

The representative load-displacement curves obtained through nanoindentation tests are shown in Fig. 6(A) for all three groups which reveal that under the same load of 1000 µN, healthy group undergoes small deformation, DC group shows moderate deformation and UC group shows high deformation. The values of hardness and modulus of dorsal, intermediate and ventral layer within each group are averaged because no statistically significant difference is found between the layers. Later the mean values of hardness and reduced modulus are calculated for healthy, DC and UC groups and shown in Table 2. The percentage decrease in the modulus calculated for DC and UC group with respect to healthy group is (9.87%, p = 0.01) and (17.17%, p < 0.001) respectively as shown in Fig. 3(B). The percentage decrease in the value of hardness for DC and UC group with respect to healthy group is (14.29%, p = 0.07) and (23.81%, p < 0.001) respectively as shown in Fig. 3(B).

The layer wise analysis of hardness and modulus are also conducted for each layer individually among all three groups and presented in Table 3. The values of percentage decrease in hardness for healthy and DC group and healthy and UC group for dorsal, intermediate and ventral layer are shown in Fig. 6(B). The degradation in values of hardness is significant in each layer of UC group with respect to healthy group (dorsal 19.49% (p = 0.02), intermediate 22.18% (p = 0.01) and ventral 23.88% (p = 0.03), and the degradation is slightly higher in ventral layer as compared to dorsal layer.

**Table 3:** Hardness and Modulus values of each layer of nail plate for healthy, diabetic controlled **(**DC) and uncontrolled diabetic (UC) group obtained through nanoindentation experiment.

	Hardness (GPa)			Modulus (GPa)		
	Healthy	DC	UC	Healthy	DC	UC
Dorsal	0.219 ± 0.05	0.199 ± 0.06	**0**.**176** ± **0**.**07**	4.94 ± 0.84	4.53 ± 0.67	**4**.**13** ± **0**.**67**
Intermediate	0.200 ± 0.05	0.179 ± 0.04	**0**.**156** ± **0**.**06**	4.63 ± 0.61	4.24 ± 0.62	**3**.**86** ± **0**.**80**
Ventral	0.199 ± 0.06	0.176 ± 0.05	**0**.**151** ± **0**.**09**	4.42 ± 0.62	3.89 ± 0.43	**3**.**56** ± **0**.**74**

The bold values are representing the significant change with respect to healthy group.

Similarly, the comparison is also made for percentage decrease in modulus values, and this is shown in Fig. 6(C). The degradation in values of modulus is also significant in each layer of UC group with respect to healthy group (dorsal 16.27% (p = 0.02), intermediate 16.63% (p = 0.002) and ventral 19.38% (p = 0.001)). The results of layer wise analysis revealed that the material properties of all three layers are getting degraded almost equally due to T2D and the degradation is slightly higher in ventral layer as compared to dorsal layer.

### Biochemical analysis

#### Calcium content

The calcium content (weight %) of fingernail plate for all three groups is shown in Table 2. The percentage decrease in calcium concentration is approximately 6.3% for DC group and 75% (p < 0.001) for UC group as compared to the healthy group as shown in Fig. 3(C).

#### CML, Protein, and Disulfide bond content, and protein structure

**Figure 7:**
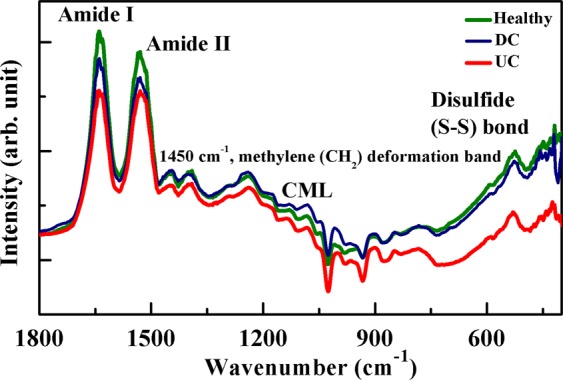
Representative FTIR spectrum of human fingernail plate showing the position of Amide I, Amide II and Disulfide (S-S) bond, CML and methylene (CH_2_) deformation band vibrations for healthy, diabetic controlled (DC), and uncontrolled diabetic (UC) groups.

The representative FTIR spectrum of fingernail plate is shown in Fig. 7 for healthy, DC and UC group. The shift in position of proteins bands (Amide I and Amide II) are observed and shown in Table 2. This shift in position indicates the altered secondary structure of proteins. The Amide I and Amide II band area are found decreased (Table 2), and the percentage decrease in the Amide I content for DC group is 22.2% (p = 0.36) and for UC group is 46.7% (p = 0.002) with respect to healthy, and Amide II content is 25% (p = 0.35) for DC group and UC group is 50% (p = 0.001) with respect to healthy as shown in Fig. 3(C). These results indicate that the overall protein content gets decreased due to T2D.

The disulfide bond content is high in healthy, moderate in DC and small in UC group as shown in Table 2. The percentage decrease in disulfide bond content for DC group is 30.6% (p = 0.05) and for UC group is 66.3% (p < 0.001) with respect to healthy as shown in Fig. 3(C), which indicates that the overall disulfide bond content is decreased due to T2D.

**Figure 8:**
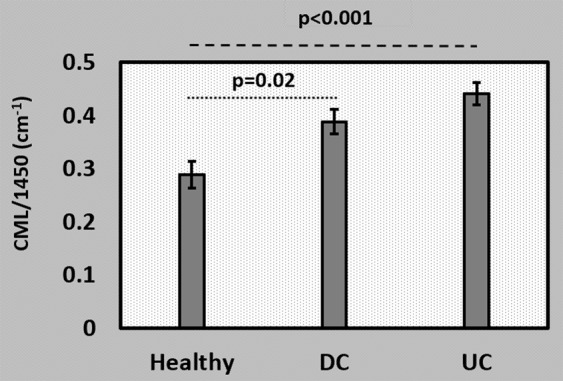
AGE measurement in the human fingernail plate through FTIR spectroscopy showed higher level of relative carboxymethyl-lysine (CML) (~1450 cm^−1^) content in diabetic controlled (DC) and uncontrolled diabetic (UC) groups.

The relative content of CML (AGE) was calculated for all three groups as shown in Fig. 8. The percentage increase in relative CML content for DC group is 34.7% (p = 0.02) and UC group is 53.1% (p < 0.001) with respect to healthy.

The above results are validated as per the published studies, which reported that the shift in peak position and/or increase in bandwidth is associated with altered proteins secondary structure^30,31^. Also, the peak intensity and/or area under the curve implies the concentration of that particular protein which is associated with that peak^30^.

## Discussion

In the present study, we showed that the T2D is affecting the fingernail plate quality by changing its structural, material and biochemical properties. We hypothesized that the main reason for degrading the nail plate quality is prolonged hyperglycemia which increases the accumulation of AGEs into the nail matrix. Later it is observed that the relative CML content is high in DC group and higher in UC group as compared to healthy. The AGEs react irreversibly with amino acid residues of peptides or proteins of nail keratin and form protein adducts (protein – AGE) or protein crosslinks (AGE-protein-AGE)^16^. The continuous AGEs accumulation is further associated with prolonged Endoplasmic Reticulum (ER) stress^32,33^. Also, the stressed ER can increase the amount of misfolded proteins^32^ in the nail matrix. Both this phenomenon ultimately leads to an alteration in ER homeostasis, which causes: 1) reduction of disulfide bonds 2) protein misfolding due to lack of chaperones 3) Ca^+2^ depletion^34^.

To maintain the ER homeostasis, the unfolded protein response (UPR) pathway is get activated through IRE1α, ATF6 and PERK stress sensors. This pathway aims to reduce the ER stress by 1) reducing the new protein synthesis to prevent the overloading of the organelle, 2) increase the amount of ER chaperones to aid the protein folding, 3) to remove the misfolded proteins from the ER and degrade the misfolded proteins in the proteasome. In the case of continuous AGEs accumulation (prolonged ER stress), the above mechanism fails to restore the ER homeostasis and leads to cellular dysfunction and cellular apoptosis^34^. This cellular dysfunction and cellular apoptosis results in increased protein misfolding (altered secondary structure of proteins) and decreased overall protein synthesis (decreased Amide I, Amide II and disulfide bond content) respectively.

These results are consistent in comparison to previous studies which have also been reported that the accumulation of AGEs is responsible for altered secondary structure of protein and decreased total protein content in diabetic rat skeletal Soleus (SOL) muscles^31^ and protein denaturation, abnormal collagen synthesis and altered collagen structure in bone^35^. The reduction in disulfide bond content has been reported for osteoporotic patients fingernail as compared to the healthy due to a reduction in total cystine content^36^ and in diabetic nail the disulfide bond gets cleaved and forms the alkyl thiolated structure^11^. The reduction in cystine content and the reduction in disulfide bond content are one and the same because the cystine molecules are made only with the help of disulfide bonds between cysteine molecules. The nail keratin is rich in cystine content, and the altered cystine content (or disulfide content) is primarily responsible for the change in the structural integrity of the nail keratin. It results in microstructural deformation in the form of increased surface roughness and decreased material properties.

We have also observed that the calcium concentration is compromised in the UC group as compared to the other two groups. The reason for calcium depletion can be explained by ER stress-mediated apoptotic pathway^37^. As the ER plays the key role in maintaining the Ca^+2^ signaling by storing and secreting the Ca^+2^ (cytosol to intra-ER and vice versa), it controls several calcium-dependent cell processes such as organogenesis, stress responses, transcriptional activity, and apoptosis. The prolonged ER stress induces the calcium depletion in the ER by activating the calcium release channel inositol 1,4,5-triphosphate receptor (IP_3_) which releases the Ca^+2^ into the cytoplasm. This increased calcium enters into mitochondria, which induces cytochrome *c*-mediated cell apoptosis and the overall calcium content gets decreased in the tissue. It has been reported that the indirect effect of hyperglycemia is associated with calciuria, i.e., continuous removal of calcium from the bone which leads to rapid bone loss^38^. Consequently, it was suggested that the nail mineral content might be a predictor of bone mineral metabolism^39^. We have also observed that the loss in calcium, increased porosity, and the decreased hardness are closely associated. Our result of depletion in calcium concentration is consistent with previous studies which have been found the inferior calcium concentration in fingernail and toenail^39^, and decreased modulus and hardness of fingernail in osteoporotic cases^40^.

The nail plate quality is degrading due to T2D. Still, the severe complications such as fracture or tearing are usually not observed in the diabetic nail. This is because the accumulation of AGE varies in the nail plate as it grows and gets replaced completely in 3–6 months’ time^41,42^. On the other hand, the continuous accumulation of AGE’s play a significant role in tissues with longer life spans such as bone as it leads to fragile bone fractures^20^. In order to use the finger nail as a new avenue to assess the bone health, we have investigated the relation between bone (type I) collagen and nail keratin. The collagen and keratin are two fibrous structural proteins produced by osteoblasts and keratinocytes respectively^29^. Both of these proteins undergo post-translational modifications in ER and in that the important modification is the formation of disulfide bond (S-S) between the cysteine molecules to get the structural stability of these proteins. In bone, the disulfide bonding is helpful for early bone formation as well as to provide strength during its matured phase whereas in nail, it provides structural integrity to nail plate. The disulfide bonding is important for the stability of noncollagenous multifunctional bone proteins named Osteonectin and the family of transforming growth factor-B (TGF-B) proteins too^43,44^. Both collagen and keratin express the vibrations of Amide bands and disulfide bonds in the spectral region (Raman and FTIR) of 1200–1800 cm^−1^ and 500–550 cm^−1^ respectively^45^. Furthermore, the adverse effect of prolonged ER stress is also reflected on osteoblasts^33^ and keratinocytes^46^, which includes proteins misfolding, reduction in proteins, calcium, and disulfide bond content, cellular dysfunction and cellular apoptosis.

Altogether, the authors proposed that the adverse effect of T2D is also reflected in the keratinized epithelial tissue such as fingernail plate quality. T2D alters the secondary structure of the protein, total content of disulfide bond, calcium, and the protein itself. It also causes the degradation in structural and mechanical properties of the fingernail plate. Comparing the results with the available literature on the diabetic bone; it can be stated that T2D similarly affects nail and bone quality through AGEs accumulation and ER stress. Authors suggest that the fingernail seems to be a new avenue to assess the diabetic bone quality.

## Material and Methods

### Sample collection

**Figure 9:**
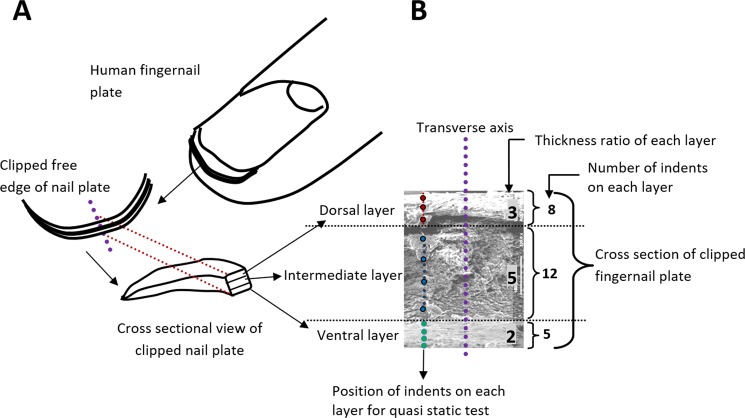
(**A**) Schematic view of free edge and clipped fingernail plate, (**B**) Pictorial representation of nanoindentation study describing indentation locations on the cross-section of fingernail plate.

The clipped fingernail plate samples Fig. 9(A) were collected from forty-five (N = 45) patients suffering from T2D for more than five years (based on patient’s clinical records) and randomly selected thirty (N = 30) healthy volunteers. The patients suffering from comorbidity diseases such as renal dysfunction, primary and secondary hyperparathyroidism, osteoporosis, unexplained elevated ALP (alkaline phosphatase) and fungal infection in fingernail were excluded from this study. The age group of the study population was 40 years and above (≥40 years). Nail plate samples were collected from a distal part of right-hand middle finger, 2–4 mm in width, using nail clipper. Collected nail samples were sectioned into 5–6 small pieces so that it can be utilized for different characterization techniques. Later the samples were transferred into sample bags, labeled and subsequently stored at −20 °C. All experiments were conducted within one months’ time after the collection of nail samples. The clinical data of T2D patients were also recorded, along with the collection of fingernail samples. All methods were carried out in accordance with relevant guidelines and regulations. The study was approved by the institutional ethics committee (Postgraduate Institute of Medical Education and Research, Chandigarh, India). Written informed consent was obtained from each study participant.

### Sample grouping

The fingernail samples were classified into three groups based on the clinical conditions of T2D prescribed by American Diabetes Association (ADA) guidelines^8,47^. The first group was referred as uncontrolled diabetic (UC). It includes the T2D patients having HbA1c greater than or equal to 7.5%^48^. The second group was referred as diabetic controlled (DC) group, it contains T2D patients having HbA1c level less than 7.5%^48^. The third group was healthy volunteers and their HbA1c level less than or equal to 5.9%^8^. After grouping, it was found out that 25 samples belonged to UC group and 20 to DC group. The mean age and age range of the study population in healthy, DC and UC groups were 58 (42–77), 55 (40–77) and 60 (42–70) years respectively. The mean age among all the three groups are found statistically insignificant as mentioned in Table 1. The mean length of T2D (7 years) is found comparable among all the patients. All participants involved in the study belonged to North India.

### Determinants of nail quality

#### Measurement of Density

The density of all the three groups of nail samples was measured with a density kit (MS DNY 54, Mettler Toledo, Greifensee, Switzerland), integrated with an electronic balance (MS105DU, Mettler Toledo, Greifensee, Switzerland).

#### Measurement of Porosity

The structure of nail plate from each group is studied using nanoscale resolution X-ray computed tomography (Phoenix Nanotom S, GE Sensing and Inspection Technologies), equipped with a high power nano focus tube with a Molybdenum target. Projection images on a CCD camera are obtained with a resolution of 2 µm for porosity analysis and 10 µm to study density variation among all three nail groups. The images are stored as TIFF files. Indexed grey values are obtained in a 16-bit format which varies between 12000 to 27000 for nail samples. The ScanIP software is used to evaluate the porosity present in the nail samples. The porosity is calculated using the formula given in Eq. (1)^49^.

1 $${\rm{Porosity}}=({\rm{volume}}\,{\rm{of}}\,{\rm{pores}}/{\rm{total}}\,{\rm{volume}}\,{\rm{of}}\,{\rm{nail}}\,{\rm{plate}}\,{\rm{sample}})\times \mathrm{100} \% $$

### Surface morphology and Calcium content

The surface morphology of the nail plate (dorsal phase and ventral phase) samples were studied through scanning electron microscope (JEOL JSM-6610LV). To make the samples conducting; a thin layer of platinum was coated using an ion sputter coating technique. Samples were observed with the secondary electron (SE) mode at a 10 kV accelerating voltage. The cross-sectional image (after tearing the nail plate sample perpendicular to its side of growth) of the nail plate is also captured through SEM and shown in Fig. 9(B).

The calcium content was studied with Energy Dispersive X-ray Spectroscopy (EDXS) using a Bruker XFlash 6I30 detector integrated with a scanning electron microscope (JEOL JSM-6610LV).

### Surface roughness

To quantify the surface roughness for the clipped dorsal phase of fingernail plate, Bruker multimode-8 Atomic Force Microscope (AFM) equipped with a silicon tip was used in contact mode. The samples were fixed from ventral side on a small disk with the help of low viscosity cyanoacrylate adhesive and the area of 10 µm × 10 µm was scanned for dorsal phase of each group.

### Material properties

The cross sections of fingernail plates were embedded in epoxy and epoxy takes 2 hours to get cured. After curing, the samples were grinded (Buehler Eco Met 250 grinder and polisher) with abrasive papers of 320, 400 and 600 grit size under the water cooling condition and polished with diamond solutions of particle sizes of 9, 6, 3, 1, 0.5 and 0.25 µm. At last the samples were sonicated for 10 minutes and then nanoindentation experiment was carried out.

Nanoindentation tests were performed using a TI-950 Tribo Indenter (Hysitron Inc., Minneapolis, MN) with Berkovich pyramidal tip, having an included angle of 142.3° and tip radius of ~150 nm. Locations for indents were identified using an optical microscope integrated with nanoindentation system and the tests were performed at room temperature.

A peak load of 1000 µN was applied on the cross-section of the fingernail plates. A load function consisting of a ten-second loading to peak force segment, followed by thirty-second hold and a ten-second unloading segment^50^ was used. Twenty-five (25) indents were performed parallel to the transverse axis on nail plate samples. The nail plate is composed of three histological layers; dorsal is the upper layer, intermediate is the middle layer and lower one is the ventral layer^51–55^. Depending on the layer wise (dorsal: intermediate: ventral) thickness ratio (3: 5: 2) of the fingernail plate^56,57^, we have got eight (8) indents on dorsal layer, twelve (12) indents on intermediate layer and five (5) indents on ventral layer as shown in Fig. 9(B). The load-displacement curves obtained in these indentation tests were analyzed to determine the reduced modulus (*E*_*r*_) and hardness (*H*) by using the method of Oliver and Pharr (OP)^58,59^.

### CML, Protein and disulfide bond content

**Figure 10:**
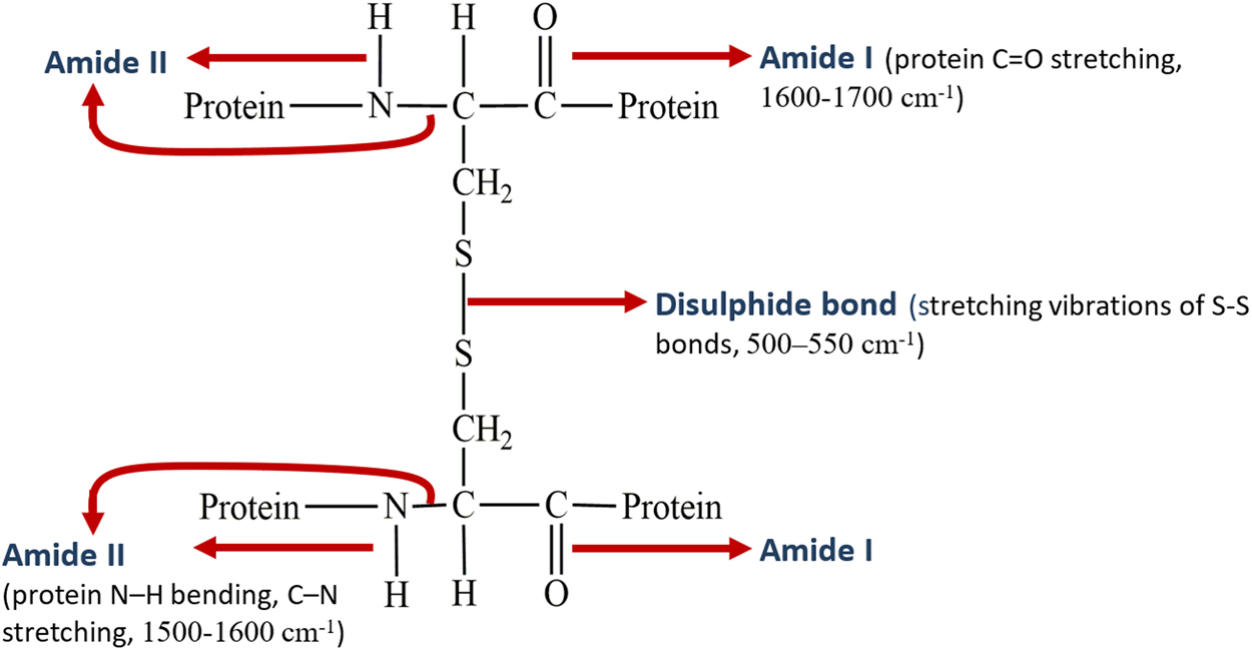
Position of Amide I, Amide II and disulfide bond in the principal keratin structural unit of fingernail plate.

The position of Amide I (protein C = O stretching, 1600–1700 cm^−1^), Amide II (protein N–H bending, C–N stretching, 1500–1600 cm^−1^), and disulphide bond (stretching vibrations of S-S bonds, 500–550 cm^−1^) in the principal keratin structural unit of nail plate is shown in Fig. 10. The FTIR spectra were recorded to study the macromolecular vibrations of above-mentioned parameters with the help of Bruker IFS 66 v/S FTIR spectrophotometer in Attenuated Total Reflectance (ATR) mode under the constant pressure in the spectral region of 4000 to 400 cm^−1^. After recording the spectra, the peak position (local maximum method), peak intensity, and area under the curve were calculated with OriginPro 8 (OriginLab, Northampton, MA) software. The mean values for the peak positions, band area of Amide I and Amide II^31,60^, and intensity of disulfide bond were calculated for each group. The intensity of the disulfide bond was measured with respect to methylene (CH_2_) deformation band at 1450 cm^−1^ ^36^.

The mean integrated area ratio (relative content) of one AGE, 1150 cm^−1^ (CML)/1450 cm^−1^ was also calculated for all the three groups. The relative CML content was derived based on previous published protocol^14,15^.

### Statistical Analysis

All the analyses were performed using SPSS 21.0 software. The normality of the data distribution was evaluated by Kolmogorov- Smirnov test^10^. A multivariate ANOVA with Tukey HSD post hoc was used to make all comparisons^45,61^. A confidence level of p < 0.05 implies a statistical significance between the groups. Results are reported as the mean ± SEM with *^,^** and *** denoting p < 0.05, p < 0.01 and p < 0.001 respectively.

## Data Availability

The datasets generated during and/or analyzed during the current study are available from the corresponding author on reasonable request.
